# The common variants implicated in microstructural abnormality of first episode and drug-naïve patients with schizophrenia

**DOI:** 10.1038/s41598-017-10507-7

**Published:** 2017-09-18

**Authors:** H. Y. Ren, Q. Wang, W. Lei, C. C. Zhang, Y. F. Li, X. J. Li, M. L. Li, W. Deng, C. H. Huang, F. Du, L. S. Zhao, Y. C. Wang, X. H. Ma, X. Hu, T. Li

**Affiliations:** 1 Mental Health Center and Psychiatric Laboratory, State Key Laboratory of Biotherapy, West China Hospital, Sichuan University, Chengdu, Sichuan PR China; 2 Brain Research Center, West China Hospital, Sichuan University, Chengdu, Sichuan PR China; 3 Psychotic Disorders Division, McLean Hospital, Belmont, Massachusetts USA; 4 Department of Psychiatry, Harvard Medical School, Boston, Massachusetts USA; 5 Biobank, West China Hospital, Sichuan University, Chengdu, Sichuan PR China

## Abstract

Both post-mortem and neuroimaging studies have identified abnormal white matter (WM) microstructure in patients with schizophrenia. However, its genetic underpinnings and relevant biological pathways remain unclear. In order to unravel the genes and the pathways associated with abnormal WM microstructure in schizophrenia, we recruited 100 first-episode, drug-naïve patients with schizophrenia and 140 matched healthy controls to conduct genome-wide association analysis of fractional anisotropy (FA) value measured using diffusing tensor imaging (DTI), followed by multivariate association study and pathway enrichment analysis. The results showed that one intergenic SNP (rs11901793), which is 20 kb upstream of *CXCR7* gene on chromosome 2, was associated with the total mean FA values with genome-wide significance (*p* = 4.37 × 10^−8^), and multivariate association analysis identified a strong association between one region-specific SNP (rs10509852), 400 kb upstream of *SORCS1* gene on chromosome 10, and the global trait of abnormal WM microstructure (*p* = 1.89 × 10^−7^). Furthermore, one pathway that is involved in cell cycle regulation, REACTOME_CHROMOSOME _MAINTENANCE, was significantly enriched by the genes that were identified in our study (*p* = 1.54 × 10^−17^). In summary, our study provides suggestive evidence that abnormal WM microstructure in schizophrenia is associated with genes that are likely involved in diverse biological signals and cell-cycle regulation although further replication in a larger independent sample is needed.

## Introduction

Schizophrenia is a chronic mental disorder that affects about 1% of the population all over the world, causing a heavy burden to the family and society^1^. Although family and twin studies showed that schizophrenia has a high heritability rate of 80%, its prevalence cannot be explained by the model of monogenic disorder^2,3^. Schizophrenia is a complex illness that is caused by multiple genetic variants of small to modest effect size^4,5^. Over the last decade, there have been a number of case-control genome-wide association studies (GWAS) of schizophrenia with different sample size in different populations. Whereas some genes have been repeatedly identified, they explained only a small fraction of schizophrenia prevalence^6,7^. With the missing heritability yet to be explored, some studies tried to expand their sample size to increase the power of GWAS^8^, which, although is promising, can be limited by funding and sample source for replication. Meanwhile, it has been recognized that schizophrenia is a complex disease without reliable biomarkers and its diagnosis has been largely based on the clinical descriptions of psychiatric practitioners, which can be biased by many confounding factors, such as the personal experience of clinicians, lack of longitudinal observation *etc*.

To address such challenges, one of the possible strategies is to use quantitative traits (QTs) of schizophrenia, which lay between the genes and the clinical diagnosis, to increase the power of study^9,10^. In fact, it has been shown that complex disorder *per se* displays the feature of QT involving multiple genes, implying that disorder’s genetic liability is distributed quantitatively rather than qualitatively^11^. Indeed, studies using measurable and objective QTs as proxy phenotypes to map genetic variants of schizophrenia have come up with promising and telling findings. For instance, Potkin *et al*. used task-related blood oxygen level–dependent (BOLD) signal from functional magnetic resonance imaging (fMRI) as the QTs to map susceptible variants in GWAS of schizophrenia. They identified that a number of variants in 3 genes and chromosomal regions (*ROBO1-ROBO2*, *TNIK*, and *CTXN3-SLC12A2*) were significantly associated with BOLD signal differences in schizophrenia^12^. Dickinson *et al*. used *g* score, which is a composite score that combines 6 neuro-cognitive dimensions, as the QTs in a genome association study to detect potential genetic variants associated with schizophrenia. This approach has led to unveil the effects of *SCN2A* on the deficits in general cognitive ability in schizophrenia and the genetic directional trend was further validated by MRI scanning and mRNA expression in schizophrenia^13^. In Chinese population, Wang *et al*. chose grey matter (GM) volume as QTs to map common variants related to schizophrenia and the results showed that the variants in the *HS3ST5, TBXAS1 and PIK3C2G* genes were significantly associated with GM volume changes in hOC3vL, vermisL10 and vermisR1^14^. Wang *et al*. later used functional connectivity as the QTs in GWAS of schizophrenia and found that the *CHRM3* gene was significantly associated with functional connectivity deficits in schizophrenia^15^. These studies highlighted the efficacy of QTs as the endophenotype in exploring the pathogenesis of schizophrenia.

Diffusion tensor imaging (DTI) is a noninvasive neuroimaging technique that yields information about the microstructure of brain white matter (WM) and the phenotype has been found to be heritable^16,17^. Moreover, abnormal WM microstructure has been repeatedly identified in both first-episode and chronic schizophrenic patients^18,19^. According to the study by Nesvag *et al*., WM abnormality was also related to the response to antipsychotic treatment^20^. Although there were several genetic studies of microstructure WM abnormality in schizophrenia^21,22^, no GWAS of schizophrenia using WM measures as QTs has been conducted. Thus, our study was carried out with the following aims: firstly, to investigate WM microstructure deficits in first-episode, drug-naïve patients with schizophrenia; secondly, to map common variants/genes underlying the microstructure deficits in patients with schizophrenia; finally, to annotate biological pathway enriched by mapped common variants/genes which are implicated in WM microstructural abnormality in patients with schizophrenia.

## Materials and Methods

### Participants

One hundred first-episode patients with schizophrenia were recruited from the West China Hospital of Sichuan University, and 140 demographically matched healthy controls without family history of mental disorders (recruited from the local neighborhood) were included in the study. Patients were interviewed with Structured Clinical Interview for Diagnostic and Statistical Manual of Mental Disorders (SCID, Patient version) by trained psychiatrists, and the diagnosis of schizophrenia was made based on criteria in Diagnostic and Statistical Manual of Mental Disorders, Fourth Edition (DSM-IV). Healthy controls were screened with SCID (Non-patient version) to ensure the absence of psychiatric illness. Patients were followed up for at least six months in order to confirm the diagnosis of schizophrenia. All patients did not use any antipsychotic medication at the time of clinical and MRI scanning. Both patients and controls were excluded if they had one of following conditions: (1) organic cerebral diseases; (2) neurological diseases; (3) severe physical diseases; and (4) axis I and II diagnosis other than schizophrenia according to DSM-IV criteria. Informed written consent was obtained from all participants after explaining the purpose and procedure of the study. This study was approved by the Institutional Review Broad (IRB) of West China Hospital, Sichuan University and all the procedures of sample collection and data analysis described here are in strict accordance with relevant guidelines and regulations.

### MRI data acquisition and preprocessing

Whole brain diffusion-weighted images were recorded along 15 gradient directions (b = 1000 s/mm^2^, number of excitations = 2) together with one unweighted (b = 0) image (42 images in total). Each image was acquired using a single-shot spin echo planar imaging (EPI) sequence [repetition time (TR) = 10000 ms, echo time (TE) = 70.8 ms, slice thickness = 3.0 mm with no gap, field of view = 240 mm^2^, matrix size = 256 × 256, voxel resolution = 0.94 × 0.94 × 3 mm^3^]. Two experienced neuroradiologists qualitatively inspected the raw MRI data. No gross abnormalities were observed for any subject. DTI data were preprocessed using the FMRIB’s Diffusion Toolbox (FDT) within FSL (http://www.fmrib.ox.ac.uk/fsl). The preprocessing included the following steps: (1) The effects of head motion and image distortion caused by eddy currents were corrected by applying an affine alignment to register all other diffusion images to the b0 images in the original DTI data. (2) The non-brain tissue and background noise were removed from b0 image using BET^23^. The diffusion tensor for each voxel was estimated by the multivariate linear fitting algorithm, and the tensor matrix was diagonalized to obtain its three pairs of eigenvalues (L1, L2, and L3) and eigenvectors. (3) The voxel-wise value of fractional anisotropy (FA) was calculated for each subject. Finally, (4) FA maps of all subjects were normalized to a 2 × 2 × 2 mm^3^ Montreal Neurological Institute standard space (MNI152) and smoothed with a 6 mm full-width at the half- maximum Gaussian kernel.

### Genotyping, quality control and imputation

The genotype data has been used by our previous studies and the details of genotyping and quality control have been published elsewhere^14,15^. In brief, DNA extracted from whole blood sample was genotyped using HumanOmniZhongHua-8 BeadChip (Illumina, San Diego, CA) and the raw genotype data underwent systematic quality control steps (QCs) including missing rate per genotype (< 5%) and per individuals (< 3%), minor allele frequency (MAF, < 0.01), Hardy-Weinberg equilibrium tests (﻿﻿﻿﻿*p﻿* < 0.001), cryptic relatedness and heterozygosity rate. In total, 109,923 SNPs and four individuals (two patients and two healthy controls) were excluded for not meeting the criteria of quality controls. The steps described above were implemented in PLINK1.07^24^. Furthermore, the standard pipeline was used to detect outliers of population stratification by using EIGENSTART^25^ with the first two components of PCA included as covariates in the subsequent analyses.

Using IMPUTE2^26^, and with the 1000 Genomes Project phase I dataset as a reference, we implemented imputation of the missing genotypes based on pre-phased haplotypes from SHAPEIT2^27^. In addition to same quality control steps (missing rate per genotype and per individuals, MAF and Hardy-Weinberg equilibrium tests), we excluded the imputed genotypes with INFO less than 0.6. After quality control, 7 patients and 6 controls were excluded due to low genotyping quality and cryptic relatedness.

### Statistical analysis

#### Comparison of FA values between patients and healthy controls

The voxel-wise comparisons of FA maps were performed using ANCOVA in SPM (Statistical Parametric Mapping) with age and sex being included as covariates. We reported the results at q < 0.01 after multiple corrections using the false discovery rate (FDR) method.

#### Univariate QT analysis and functional annotation

In order to detect genetic loci associated with FA values in brain regions identified from above, a mixed linear model in MixABEL of ABEL packages^28^ was used to test association of FA values in the brain regions showing significant inter-group difference with the interaction effect of genotype × group under the assumption of additive dosage effect; controlling for age, sex and﻿ the first two components from population PCA﻿﻿.﻿ ﻿﻿Following the QT analysis, the top associated SNPs were annotated in terms of histone modification^29^, regulatory motif alterations^30^ and Regulome DB scores using both Haploreg^31^ and Regulome DB^32^.

In order to detect potential effect of gene-gene interaction (epistasis) on the QTs in our study, an analysis using model-based multifactor dimension reduction (MB-MDR) was conducted with two order combination of the top SNPs identified from univariate QT analysis as epistasis variables and covariates such as age, gender and main effect of single SNP being controlled for. In brief, MB-MDR consists of 3 steps: (1) an association test for each multilocus cell with the phenotype: a logistic regression for binary traits, a linear regression for was performed. Each genotype cell is then assigned to one of three categories, high risk (H), low risk (L) or no evidence (0), as a result of the association test with a liberal threshold of 0.1 for assigning the genotype to a risk category, H or L. (2) Two new association analysis, one for each risk category, high and low, on the outcome variable was implemented, with the corresponding unadjusted *p* value for each test (PH and PL) being generated. The minimum between PH and PL is chosen as minimum P value. (3) The significance of the specified models was decided through 1000 permutations on the maximum Wald statistic from step 2^33^.

#### Multivariate association study

To identify the shared genetic effects within multiple regions of abnormal white matter microstructure in schizophrenia, we conducted a multivariate genotype-phenotype analysis using efficient multivariate genotype-phenotype analysis for genome-wide association studies (Trait-based Association Test that uses Extended Simes procedure, TATES)^34^. This analysis presents a global trait-based *p* value (P_T_) by combining the *p* values derived from standard univariate GWAS and correcting for the observed correctional structure between the phenotypes (i.e. multiple abnormal white matter microstructure in the present study). The global trait-based results from TATES were then used to conduct gene-based testing using Gene-Based Association Test Using Extended Simes Procedure (GATES)^35^ in Knowledge-based mining system for Genome-wide Genetic studies (KGG) platform. After mapping SNPs onto genes according to the gene coordinate information from NCBI (SNPs within 5 kilobase pairs of each gene were also assigned into the gene) and weighted by LD coefficient between SNPs, GATES rapidly combine the *p* values of SNPs within a gene and generate a gene-based *p* value by dividing independent *p* values of effective number among the SNPs within a gene by independent *p* values of the effective number among the top SNPs.

#### Pathway enrichment analysis using KGG

In order to further understand and interpret the association results from our study, we explored the biological pathway enriched by the genes associated with WM microstructure abnormality using hybrid set-based test (HYST) combining the extended Simes’ test and scaled chi-square test in KGG platform^36^. To reduce type I error, a Bonferroni adjustment for multiple testing was conducted using a significant threshold of 0.05/N, where N = number of gene sets in GSEA (gene set enrichment analysis) pathway database (0.05/17779 ~ = 2.81×10^–6^).

## Results

### Demographic characteristics

All participants were Han Chinese. As shown in Table 1, there was no significant difference observed in age, years of education and gender between the patients and healthy controls. The median duration of illness for patients was 5.5 months (SD: 26.7). All patients were drug-naïve at the time of clinical evaluation and MRI scanning.

**Table 1:** Summary of demographic and clinic characteristics of subjects.

	Patients (93)	Healthy controls (134)	Statistics	*p*
Gender (% Male)	44.29	55.71	0.36	0.71
Years of education	10.60 ± 5.12	11.86 ± 5.42	−1.46	0.14
Age	21.57 ± 12.85	21.08 ± 10.78	0.38	0.7
Positive scale	25.64 + 10.08	N/A	N/A	N/A
Negative scale	18.69 + 9.78	N/A	N/A	N/A
General scale	45.74 + 20.76	N/A	N/A	N/A
Duration of untreated psychosis (month)	5.5 ± 26.72	N/A	N/A	N/A
ACC-L	0.24 ± 0.012	0.25 ± 0.014	45.09	<0.001*
ACC-R	0.21 ± 0.012	0.22 ± 0.014	33.1	<0.001*
IPC-L	0.22 ± 0.019	0.24 ± 0.019	23.01	<0.001*
PCC-L	0.38 ± 0.020	0.40 ± 0.020	32.63	<0.001*
PCC-R	0.42 ± 0.030	0.45 ± 0.020	37.01	<0.001*
Total mean FA	0.36 ± 0.020	0.38 ± 0.019	47.22	<0.001*

ACC_L: Left anterior cingulate cortex; ACC-R: Right anterior cingulate cortex; IPC-L: Left inferior parietal cortex; PCC-L: Left posterior cingulate cortex; PCC-R: Right posterior cingulate cortex; *p values were adjusted using FDR.

### Comparison of white matter microstructure in patients with schizophrenia and controls

**Figure 1:**
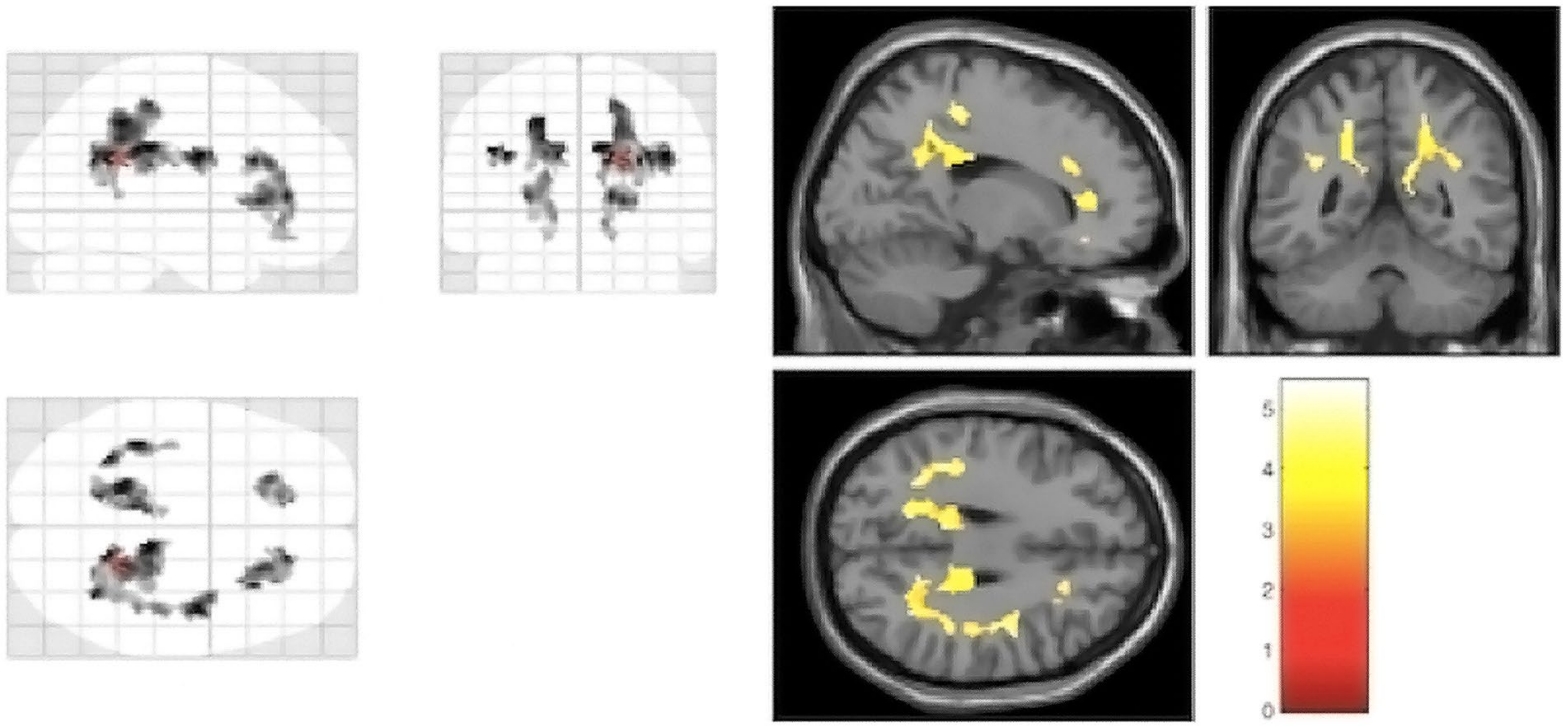
DTI analysis revealing reductions of fractional anisotropy (FA) in schizophrenia patients compared to healthy controls in left anterior cingulate cortex, right anterior cingulate cortex, left inferior parietal cortex, left posterior cingulate cortex and right posterior cingulate cortex. Significant voxels (*p* = 0.05, corrected; voxel threshold k = 100 voxels) are shown as maximum intensity projection (left panel) and single subject T3-weighted image (right panel).

Two hundred and twenty-seven subjects (93 cases and 134 controls) had been scanned by MRI successfully. As shown in Figure 1, patients had reduced FA values in 5 brain regions, i.e. left anterior cingulate cortex (ACC-L), right anterior cingulate cortex (ACC-R), left inferior parietal cortex (IPC-L), left posterior cingulate cortex (PCC-L) and right posterior cingulate cortex (PCC-R), in comparison with healthy controls. The differences survived after FDR multiple corrections. The total mean FA value, which was calculated by combining and averaging FA values of all the brain regions, was significantly lower in patients compared to healthy controls. Therefore, we included total mean FA values along with FA values of 5 brain regions as QTs in subsequent association analysis.

### GWAS analysis of WM microstructural abnormalities in schizophrenia

**Figure 2:**
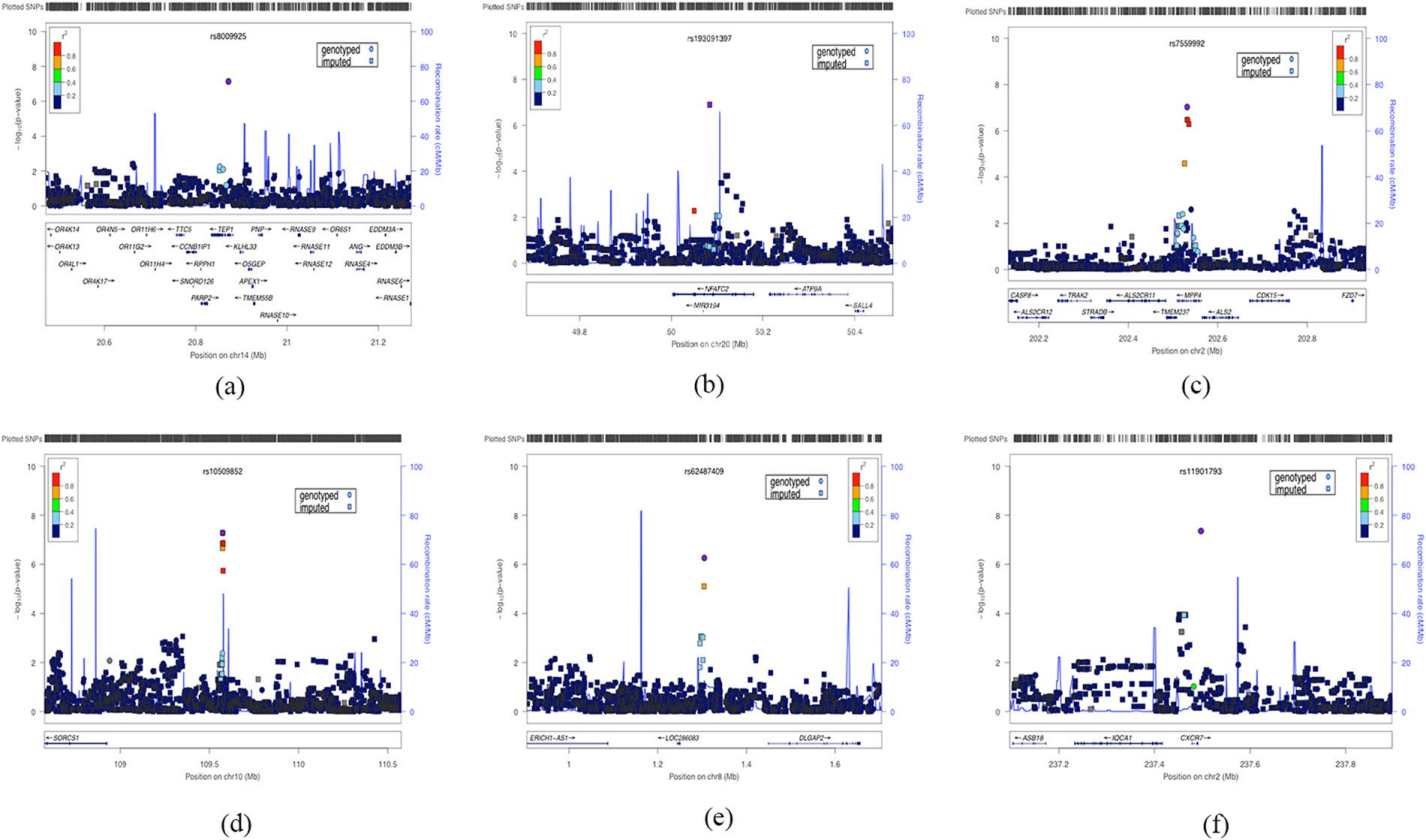
The regional association plot using web-interface of LOCUSZOOM, (**a**) ACC-L ; (**b**) ACC-R ; (**c**) IPC-L ; (**d**) PCC-L ; (**e**) PCC-R ; (**f**) total mean FA values.

In total, 227 subjects with high-quality genotypes (6,055,918 SNPs) remained and there was no notable population stratification within our sample. Following univariate association analysis, we found an association of a genotyped SNP (rs11901793) within 6.2 kb upstream of *CXCR7* gene on chromosome 2 with the total mean FA value that passed the genome-wide threshold significance (*p* = 4.37 × 10^–8^). The other genotyped SNP, rs10509852, which is located in the intergenic region within 400 kb downstream of *SORCS1* on chromosome 10 showed the second strongest signal after association analysis with FA values of PCC-L (*p* = 5.21 × 10^−8^). The regional plottings of SNP with the strongest association for each of the six traits were displayed in Figure 2 and the summary of results are shown in Supplementary Table 1. Accordingly, our power was 76% for an effect size of 0.41, at an allele frequency of 0.42^37^. Besides, gene-gene interaction analysis using MB-MDR observed a significant high-risk effect of interaction between rs10509852 (SORCS1) and rs11901793 (CXCR7) on the FA values in PCC-L in both cases (Wald statistic = 14.45, permutation *p*﻿ = 0.008) and controls (Wald statistic = 14.38, permutation *﻿p* = 0.008) after 1000 permutations, highlighting the involvement of epistasis between these two genes in white matter microstructure in PCC-L independent of diagnosis.

Moreover, functional annotation of 6 top associated SNPs indicated that these SNPs played an important role in transcription factor binding and transcription-enhancing in the tissues related to neurodevelopment and immunology (Table 2).﻿

**Table 2:** Functional annotation of the risk SNPs associated with WM microstructural abnormalities in schizophrenia.

CHR	POS (hg19)	SNP	Chromatin states	Motifs changed	Genes	Positional	RegulomeDB
						annotation	
8	1304999	kgp5929161	Enhancer	DMRT2	20 kb 3′ of DLGAP2	NA	TF binding or DNase peak
		(rs62487409)	(ESC, iPSC)				
14	20871645	rs8009925	Enhancer	5 altered motifs	TEP1	intronic	TF binding or DNase peak
			(iPSC)				
20	50083574	rs193091397	Enhancer	AhR,CHOP::CEBPalpha	NFATC2	intronic	No Data
			(Blood & T-cell)	NRSF			
2	202531547	rs7559992	Enhancer	6 altered motifs	MPP4	intronic	Others
			(NH-A Astrocytes Primary Cells)				
10	109575094	rs10509852	Enhancer	RXR::LXR	400kb 5′ of SORCS1	NA	TF binding or DNase peak
			(ES-deriv, HSC & B-cel)				
2	237497237	rs11901793	Enhancer	5 altered motifs	6.2 kb 3′ of CXCR7	NA	TF binding or DNase peak
			(ES-deriv, Neurosph)				

### Multivariate analysis and pathway enrichment

**Figure 3:**
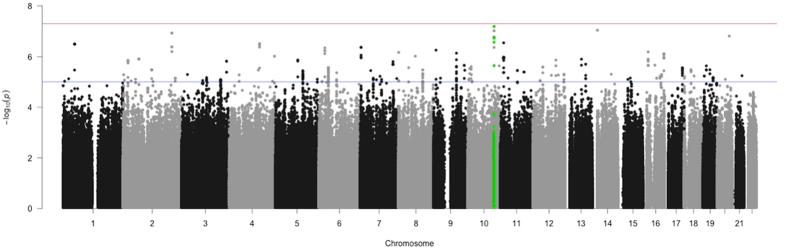
Manhattan plot of TATES test, green highlighted points are SNPs located within 400 kb of SORCS1 gene.

Besides, the same SNP, *rs*10509852, yielded the most significant global trait-based *p* value (*p* = 1.89 × 10^−7^) after combining *p* values from univariate association analysis of FA values from 5 brain regions (ACC-L, ACC-R, IPC-L, PCC-L, PCC-R) using TATES (Figure 3). Furthermore, the lambda (λ) score following association study indicated that all confounding factors regarding genotyping and population structure have been well-adjusted which is illustrated through Q-Q plot in Supplementary Figure 1. The gene-based test using GATES showed that 8 genes (Table 3) consisting of four non-coding micro-RNA genes (*miRNA-27a, miRNA24-2, LOC284454*, and *miRNA-23a*) and four protein-coding genes (*TEP1, PDZD9, MPP4, and UQCRC2*) passed the significance threshold (*p* < *0.05*), details of these genes are shown in Supplementary Table 2.

**Table 3:** Gene-based association using GATE.

Symbol	Corrected *p*	Chromosome	Start_Position	Group
TEP1	0.034	14	20833825	protein-coding gene
PDZD9	0.034	16	21995185	protein-coding gene
MPP4	0.034	20	50083574	protein-coding gene
miRNA-27a	0.034	19	13947253	non-codingRNA
miRNA24-2	0.034	19	13947100	non-codingRNA
LOC284454	0.034	19	13945329	unknown
miRNA-23a	0.034	19	13947400	non-codingRNA
UQCRC2	0.034	16	21964608	protein-coding gene

**Figure 4:**
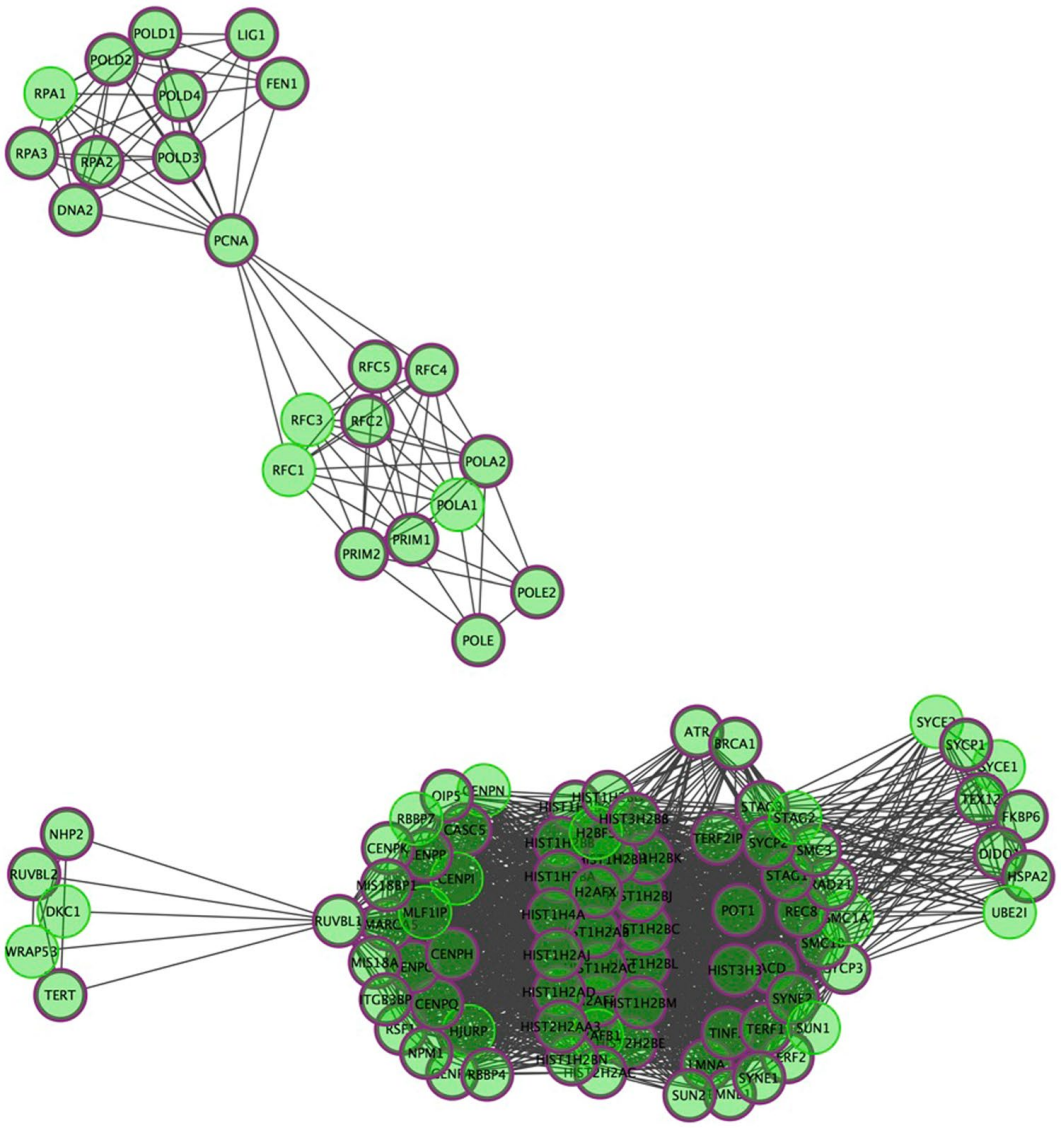
Reactome F1 network visualization of enriched pathway (**REACTOME_CHROMOSOME_MAINTENANCE**), nodes with thick purple circle in diagram were hit genes.

Furthermore, hybrid set-based test (HYST) in KGG identified a cell cycle pathway (REACTOME_CHROMOSOME_MAINTENANCE, *p*﻿ = 8.68 × 10﻿^−22^﻿) to be significantly enriched and remained significant after the Bonferroni correction for multiple comparisons (Corrected *p* = 1.54 × 10^−17^). Figure 4 displayed the Reactome F1 network and showed that out of 104 genes constituting the pathway, 38 genes have nominally significant gene-wise *p* values in our study (Details of 38 genes are displayed in Supplementary Table 2).

## Discussion

To our knowledge, this is the first study to use FA values as QTs in GWAS of schizophrenia. In our study, after inter-group comparison, FA values in 5 brain regions (ACC-L, ACC-R, IPC-L, PCC-L, PCC-R) and the total mean FA values showed a significant difference between first-episode, drug-naïve schizophrenia patients and controls after multiple corrections. Moreover, using microstructural abnormality as QTs, we also found that two variants near/within *CXCR7* and *SORCS1* genes, eight genes (four non-coding micro-RNA genes including *miRNA-27a, miRNA24-2, LOC284454* and *miRNA-23a* and four protein-coding genes including *TEP1, PDZD9, MPP4 and UQCRC2*), and one cell cycle pathway (REACTOME_CHROMOSOME_MAINTENANCE) were implicated in WM microstructural abnormality of schizophrenia in the present study.

Of the five brain regions, ACC-L, ACC-R, PCC-R, and PCC-L belong to the cingulum bundle (CB) which connects the limbic structure with frontal and temporal cortex. Previous studies have also identified the alterations of CB in schizophrenia. Kubicki *et al*. found that the mean FA values in bilateral CB in schizophrenic patients was decreased while compared to the controls^38^. Voineskos *et al*. examined the microstructural integrity of frontotemporal and interhemispheric WM tracts in schizophrenia across the adult lifespan, while younger patients had lower FA values in the right CB in relation to the younger controls, this difference disappeared in the older group of patients^39^. In addition, our study also found that FA values in the left inferior parietal lobe were significantly lower in patients than controls and the finding is consistent with those of Ardekani *et al*. and Rowland *et al*.^40,41^. It is worth noting that, patients with schizophrenia in our study showed significantly lower total mean FA values. In fact, schizophrenia has long been presumed to be the illness with comprehensive deficits of WM^42,43^ and our findings derived from the total mean FA values implies that focal WM deficit might appear based on the contextual whole-brain WM alterations.

In the present study, we found that an intergenic SNP (rs11901793) flanking *CXCR7* gene (6.2 kb 3′) was associated with the total mean FA values with genome-wide significance. *CXCR7* is a chemokine receptor which modulates cell migration in multiple biological contexts including brain development, especially in the signaling modulation of GABaergic interneurons^44–46^. It has been found in various studies that the deficits in the development of GABaergic neurons were involved in the pathogenesis of schizophrenia, especially the cognitive impairment in schizophrenia^47–49^. Moreover, an intergenic SNP (rs10509852) flanking *SORCS1* gene on chromosome 10 was associated with both (PCC-L) and the global trait. *SORCS1* is one of the transmembrane receptor family with a homologous domain to the yeast vacuolar sorting protein VPS10p (Sortilin) and it has been demonstrated to bind with the neuropeptides and neurotensins^50–52^. Intriguingly, one study showed that Sortilin played an important role in BDNF sorting that regulated the secretory pathway by interacting specifically with BDNF in a region encompassing the methionine substitution, and colocalized with BDNF in secretory granules in neurons^53^. Indeed, both BDNF and neuronal cell death have been found to be involved in the pathophysiology of schizophrenia. Our findings provided further insights into understanding the mechanism of schizophrenia, especially the one associated with these seemingly diverse biological signals. Intriguingly, the majority of the top associated SNPs in our study was found to confer their impact through enhancing the different transcription factors in tissues critical of neurodevelopment and immunology, such as derived neuronal progenitor cultured cells (ES-deriv), cortex derived primary cultured nuerosphere (ES-deriv, Neurosph) and T-cell. Such a pattern shows another piece of evidence that neurodevelopmental and immunological pathways are implicated in the pathogenesis of schizophrenia. A future study could focus on the biological function of the protein-coding sequence to which these transcription factors bind and also on the effect of environmental factors on the function of these transcription factors, especially the chromatin states.

In our gene-based analysis, we found eight genes that were significantly associated with the global trait of FA values from five correlated brain regions (ACC-L, ACC-R, IPC-L, PCC-L, PCC-R) and survived the multiple corrections after pooling effect size of all variants of each gene together. Of these eight genes, *TEP1*, telomerase-associated protein 1, is responsible for catalyzing the addition of new telomeres to chromosomes and prevents developing neurons from DNA damage-induced cell death^54,55^. *MPP4*, membrane protein, palmitoylated 4, functions as scaffold proteins that contribute to the cell polarity and organize signal transducers at the neuronal synapse membrane^56^. Although it remains unclear on the pathophysiological role of these two genes, our study provided evidence to support the involvement of these two genes in schizophrenia. Future research on the biological functions of genes should be justified. Another four genes which were significantly associated with the abnormal white matter microstructure in schizophrenia at the gene-based level encode the non-coding micro-RNA (*miRNA-27a*, *miRNA24-2*, *LOC284454* and *miRNA23a*). In recent years, micro-RNAs have gained increasing attention in the etiology of schizophrenia. In fact, over half of the miRNAs identified so far have been shown to be highly or exclusively expressed in the brain, and some of them have been found to be abnormally expressed in patients with schizophrenia^57,58^. Of interest, one of the important regulatory targets of miRNA23a gene is CXCL12, of which CXCR7 is the main receptor^59^.

Furthermore, our pathway analysis identified an enriched cell cycle pathway, REACTOME_CHROMOSOME_MAINTENANCE, which was associated with WM microstructure abnormality in schizophrenia. The previous study has shown that accurate neuronal cell cycle is one of the most critical components for neuronal development, deviation of which can cause premature cell cycle exit^60,61^. Using unbiased molecular profiling analysis, Wang *et al*. obtained skin fibroblasts from the living patients with schizophrenia and identified perturbations in cell cycle proteins. They further validated their results at expression and functional levels in the same study^62^. Moreover, for 38 genes enriched in this pathway, some of them have already been found to function in neurodevelopment and our results provided additional evidence for supporting the role of cell cycle pathway in schizophrenia, but further studies were still required to validate our present findings.

Although the results of our study are promising and reliable, we fully acknowledge that there are a few limitations. Firstly, we conducted studies in schizophrenic patients as a whole and did not further stratify the patients based on their clinical features. Although the traits related to the disease as a whole can avoid the confounding factors arisen from the subjective categorization of clinical symptomology, future studies should focus on the genetic basis of WM microstructure abnormality in different subgroups of schizophrenia. Secondly, due to the complexity of schizophrenia and modest sample size of our study, any definitive conclusions should be precluded, and future studies in a larger sample are needed to validate our findings.

In conclusion, our results suggest that WM abnormalities of schizophrenia patients mainly present in CB region and parietal cortex. These abnormalities are associated with the genes that are likely to be involved in diverse biological signals and highly enriched in the biological pathway of cell cycle. In addition, our study suggests that the strategy of using neuroimaging measures as QTs of schizophrenia could significantly improve the power of GWAS to identify the susceptible genes/pathways of schizophrenia.

## Electronic supplementary material

Supplementary information 
