# Supplementary material for: The common variants implicated in microstructural abnormality of first episode and drug-naïve patients with schizophrenia
Source: Sci Rep. 2017 Sep 18;7:11750. doi: 10.1038/s41598-017-10507-7 (PMC5603592; doi:10.1038/s41598-017-10507-7)
Supplement: Supplementary file 1 — Supplementary information [file 41598_2017_10507_MOESM1_ESM.doc]

# The common variants implicated in microstructural abnormality of first episode and drug-naïve patients with schizophrenia

HY Ren^1,2*^, Q Wang^1,2*^, W Lei^1,2^, CC Zhang^1,2^, YF Li^1,2^, XJ Li^1,2^, ML Li^1,2^, W Deng^1,2^, CH Huang^1,2^, F Du^3^, LS Zhao^1,2^, YC Wang^1,2^, XH Ma^1,2^, X Hu^4#^, T Li^1,2#^

^1^Mental Health Center and Psychiatric Laboratory, State Key Laboratory of Biotherapy, West China Hospital, Sichuan University, Chengdu, Sichuan, PR China

^2^Brain Research Center, West China Hospital, Sichuan University, Chengdu, Sichuan, PR China

^3^Psychotic Disorders Division, McLean Hospital, Belmont, Massachusetts; Department of Psychiatry, Harvard Medical School, Boston, Massachusetts, US.

^4^Biobank, West China Hospital, Sichuan University, Chengdu, Sichuan, PR China

^*^These authors contributed equally to this work

#Corresponding authors:

Dr T Li, Mental Health Center and Psychiatric Laboratory, State Key Laboratory of Biotherapy, West China Hospital, Sichuan University, Chengdu, Sichuan, PR China. Email: [litaohx@scu.edu.cn](mailto:litaohx@scu.edu.cn)

Dr X Hu, Biobank, West China Hospital, Sichuan University, Chengdu, Sichuan, PR China.

Email:hxxhu@163.com

Supplementary Table 1 The most significant association of common variants with abnormality of white matter microstructural regions in patients with schizophrenia

| CHR | POS (hg19) | SNP | Genotype | MAF | Chisq | beta_SNP: group | mean_SNP: group | *p*-value | Brain-region |
| --- | --- | --- | --- | --- | --- | --- | --- | --- | --- |
| 8 | 1304999 | kgp5929161 | Genotyped | 0.06 | 28.82 | 0.06 | 0.07 | 5.52E-07 | PCC_R |
| 14 | 20871645 | rs8009925 | Genotyped | 0.33 | 32.85 | 0.04 | 0.28 | 7.35E-08 | ACC_L |
| 20 | 50083574 | rs193091397 | Imputed | 0.01 | 31.77 | 2.83 | 0 | 1.26E-07 | ACC_R |
| 2 | 202531547 | rs7559992 | Genotyped | 0.21 | 32.36 | -0.03 | 0.21 | 9.38E-08 | IPL_L |
| 10 | 109575094 | rs10509852 | Genotyped | 0.24 | 33.54 | -0.04 | 0.41 | 5.21E-08 | PCC_L |
| 2 | 237497237 | rs11901793 | Genotyped | 0.23 | 33.89 | -0.02 | 0.3 | 4.37E-08 | Total-mean FA |

Abbreviations: CHR, chromosome; POS, physical position; SNP, single-nucleotide polymorphism; PCC_R, right posterior cingulate cortex; ACC_L, left anterior cingulate cortex; ACC_R, right anterior cingulate cortex; IPL_L, left inferior parietal lobule; PCC_L, left posterior cingulate cortex.


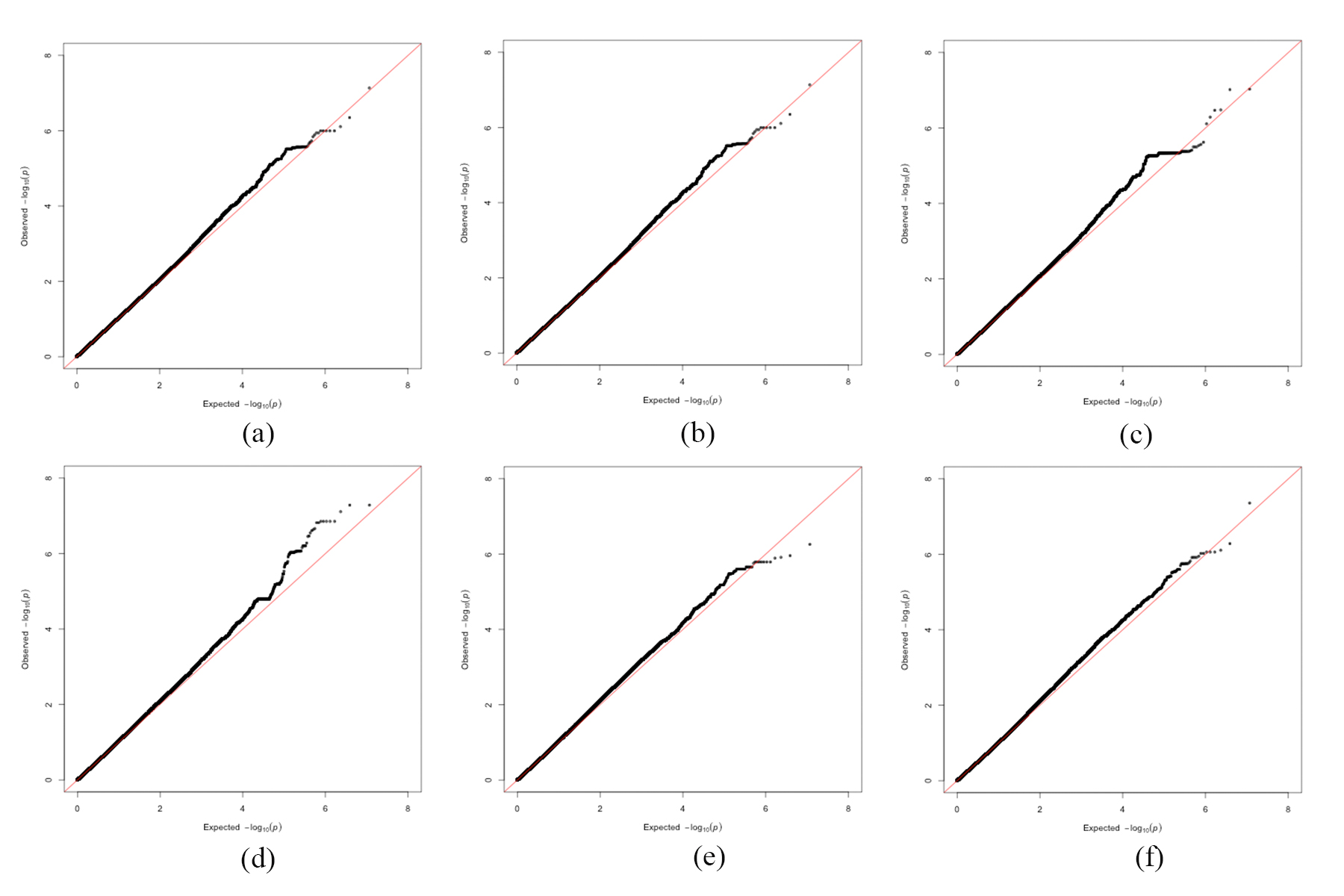


Supplementary Figure 1: Quantile-quantile plot for GWAS findings with FA values in five brain region (a left anterior cingulate cortex; b right anterior cingulate cortex; c left inferior parietal cortex; d left posterior cingulate cortex; e right posterior cingulate cortex) and total mean FA values(f) as QTs. The plot shows actual vs expected −2log(e)***p*** for FA values in cases and control individuals. −2Log(e)***p*** follows a χ2 distribution (df = 2) and can be used for statistical inference. Points above the horizontal line indicate an enrichment of low *p* values beyond what would be expected by chance.

Supplementary Table 2 Summary of SNPs located in 8 genes associated with WM microstructural abnormality in schizophrenia at gene level

| **Gene** | **GENE P** | **Chromosome** | **Start_Position** | **Length** | **Group** | **SNP** | **Position** | **Feature** | **SNP *p*** |
| --- | --- | --- | --- | --- | --- | --- | --- | --- | --- |
| TEP1 | 0.034375023 | 14 | 20833825 | 47762 | protein-coding gene | rs8009925 | 20871645 | intronic | 9.11627E-08 |
| - | - | - | - |  | - | rs1760897 | 20876253 | exonic | 0.000522852 |
| - | - | - | - |  | - | rs1713439 | 20875750 | intronic | 0.000572423 |
| - | - | - | - |  | - | rs2104977 | 20838713 | intronic | 0.00074411 |
| - | - | - | - |  | - | rs35159459 | 20838398 | intronic | 0.000857175 |
| - | - | - | - |  | - | rs4981998 | 20829032 | downstream | 0.004015151 |
| - | - | - | - |  | - | rs10129957 | 20830046 | downstream | 0.00429278 |
| - | - | - | - |  | - | rs4415933 | 20830809 | downstream | 0.00429278 |
| - | - | - | - |  | - | rs7161611 | 20831406 | downstream | 0.00429278 |
| - | - | - | - |  | - | rs12323610 | 20832318 | downstream | 0.00429278 |
| - | - | - | - |  | - | rs12323412 | 20832276 | downstream | 0.00429278 |
| - | - | - | - |  | - | rs10130660 | 20829372 | downstream | 0.00429278 |
| - | - | - | - |  | - | rs10147163 | 20829438 | downstream | 0.00429278 |
| - | - | - | - |  | - | rs10148359 | 20829966 | downstream | 0.00429278 |
| - | - | - | - |  | - | 14:20877081 | 20877081 | intronic | 0.006948119 |
| - | - | - | - |  | - | rs1760903 | 20852817 | exonic | 0.006968913 |
| - | - | - | - |  | - | rs1713423 | 20860073 | intronic | 0.009933107 |
| - | - | - | - |  | - | rs1760904 | 20852029 | exonic | 0.011126741 |
| - | - | - | - |  | - | rs139071292 | 20843503 | intronic | 0.020720713 |
| - | - | - | - |  | - | rs146208574 | 20856021 | intronic | 0.021963032 |
| - | - | - | - |  | - | rs2228035 | 20871973 | exonic | 0.026661973 |
| - | - | - | - |  | - | rs4246977 | 20882591 | upstream | 0.03307982 |
| - | - | - | - |  | - | rs12882604 | 20861828 | intronic | 0.033652145 |
| - | - | - | - |  | - | rs2945461 | 20863070 | intronic | 0.03476304 |
| - | - | - | - |  | - | rs12888864 | 20857164 | intronic | 0.037107603 |
| - | - | - | - |  | - | rs35202386 | 20856479 | intronic | 0.037107603 |
| - | - | - | - |  | - | 14:20859084 | 20859084 | intronic | 0.041855195 |
| - | - | - | - |  | - | rs12895477 | 20854012 | intronic | 0.04544191 |
| - | - | - | - |  | - | rs7157027 | 20883064 | upstream | 0.045754226 |
| - | - | - | - |  | - | rs35384067 | 20858463 | intronic | 0.046371711 |
| - | - | - | - |  | - | rs872074 | 20859205 | exonic | 0.046371711 |
| - | - | - | - |  | - | rs34734346 | 20860568 | intronic | 0.046371711 |
| - | - | - | - |  | - | rs35793276 | 20861101 | intronic | 0.046371711 |
| PDZD9 | 0.034375023 | 16 | 21995185 | 17245 | protein-coding gene | rs8043580 | 22003346 | intronic | 6.55114E-07 |
| - | - | - | - |  | - | rs9934359 | 21999124 | intronic | 1.52868E-06 |
| - | - | - | - |  | - | rs6497564 | 22016637 | upstream | 5.35517E-05 |
| - | - | - | - |  | - | 16:21997551 | 21997551 | intronic | 0.000175713 |
| - | - | - | - |  | - | rs11645673 | 22011122 | intronic | 0.00114417 |
| - | - | - | - |  | - | rs4392055 | 21997834 | intronic | 0.00180098 |
| - | - | - | - |  | - | rs56036711 | 21990760 | intronic | 0.00180098 |
| - | - | - | - |  | - | rs77032576 | 21991449 | intronic | 0.00180098 |
| - | - | - | - |  | - | rs4281695 | 22014642 | upstream | 0.001909902 |
| MPP4 | 0.034375023 | 2 | 202509596 | 53824 | protein-coding gene | rs7559992 | 202531547 | intronic | 1.16286E-07 |
| - | - | - | - |  | - | 2:202541707 | 202541707 | intronic | 1.1951E-07 |
| - | - | - | - |  | - | rs199957979 | 202531454 | intronic | 4.12161E-07 |
| - | - | - | - |  | - | rs1208083 | 202532447 | intronic | 4.21235E-07 |
| - | - | - | - |  | - | rs79943002 | 202535393 | intronic | 6.4052E-07 |
| - | - | - | - |  | - | rs62193395 | 202525800 | intronic | 0.000032479 |
| - | - | - | - |  | - | 2:202526589 | 202526589 | intronic | 5.71276E-05 |
| - | - | - | - |  | - | rs17468821 | 202529514 | intronic | 0.000149512 |
| - | - | - | - |  | - | rs17384293 | 202529479 | intronic | 0.000341039 |
| - | - | - | - |  | - | rs6731576 | 202540420 | intronic | 0.000990308 |
| - | - | - | - |  | - | rs4675208 | 202513608 | intronic | 0.003729356 |
| - | - | - | - |  | - | rs4675209 | 202513615 | intronic | 0.003729356 |
| - | - | - | - |  | - | rs183340490 | 202548317 | intronic | 0.003875555 |
| - | - | - | - |  | - | rs888011 | 202521426 | intronic | 0.005029655 |
| - | - | - | - |  | - | rs10931966 | 202517674 | intronic | 0.009946169 |
| - | - | - | - |  | - | rs10931965 | 202517523 | intronic | 0.01322957 |
| - | - | - | - |  | - | rs76603204 | 202521104 | intronic | 0.015044706 |
| - | - | - | - |  | - | rs1914258 | 202545984 | intronic | 0.01506536 |
| - | - | - | - |  | - | rs4673204 | 202518374 | intronic | 0.015662994 |
| - | - | - | - |  | - | rs4675212 | 202513883 | intronic | 0.016434152 |
| - | - | - | - |  | - | rs4675211 | 202513765 | intronic | 0.016434152 |
| - | - | - | - |  | - | rs4675210 | 202513735 | intronic | 0.016434152 |
| - | - | - | - |  | - | rs4675207 | 202513277 | intronic | 0.016434152 |
| - | - | - | - |  | - | rs111275745 | 202511848 | intronic | 0.016448569 |
| - | - | - | - |  | - | rs10169154 | 202516775 | intronic | 0.016482223 |
| - | - | - | - |  | - | rs4516414 | 202516907 | intronic | 0.016482223 |
| - | - | - | - |  | - | rs140297939 | 202532854 | intronic | 0.017526072 |
| - | - | - | - |  | - | rs78263622 | 202516809 | intronic | 0.01807103 |
| - | - | - | - |  | - | rs13402805 | 202547964 | intronic | 0.018425287 |
| - | - | - | - |  | - | rs1962630 | 202518762 | intronic | 0.018770658 |
| - | - | - | - |  | - | rs2597900 | 202523347 | intronic | 0.021596784 |
| - | - | - | - |  | - | rs76810460 | 202510974 | intronic | 0.028598753 |
| - | - | - | - |  | - | rs74887695 | 202528830 | intronic | 0.028663266 |
| - | - | - | - |  | - | rs2287050 | 202555132 | intronic | 0.028738596 |
| - | - | - | - |  | - | rs3754932 | 202509814 | 3UTR | 0.033462562 |
| - | - | - | - |  | - | rs2110738 | 202509606 | 3UTR | 0.037045212 |
| - | - | - | - |  | - | rs16838004 | 202547528 | intronic | 0.03886269 |
| - | - | - | - |  | - | rs62193402 | 202550083 | intronic | 0.04023628 |
| - | - | - | - |  | - | rs2241135 | 202507232 | intronic | 0.041648339 |
| - | - | - | - |  | - | rs62193401 | 202546557 | intronic | 0.045835784 |
| MIR27A | 0.034375023 | 19 | 13947253 | 77 | non-coding RNA | rs35421192 | 13950221 | upstream | 2.31781E-06 |
| - | - | - | - |  | - | rs34597097 | 13950294 | upstream | 3.33579E-06 |
| - | - | - | - |  | - | 19:13950253 | 13950253 | upstream | 3.33579E-06 |
| - | - | - | - |  | - | rs7248169 | 13949751 | upstream | 3.38868E-06 |
| - | - | - | - |  | - | rs62122064 | 13950206 | upstream | 1.20859E-05 |
| - | - | - | - |  | - | rs1531212 | 13951830 | upstream | 0.000559151 |
| - | - | - | - |  | - | rs895819 | 13947292 | upstream | 0.002824047 |
| MIR24-2 | 0.034375023 | 19 | 13947100 | 72 | non-coding RNA | rs35421192 | 13950221 | upstream | 2.31781E-06 |
| - | - | - | - |  | - | rs34597097 | 13950294 | upstream | 3.33579E-06 |
| - | - | - | - |  | - | 19:13950253 | 13950253 | upstream | 3.33579E-06 |
| - | - | - | - |  | - | rs7248169 | 13949751 | upstream | 3.38868E-06 |
| - | - | - | - |  | - | rs62122064 | 13950206 | upstream | 1.20859E-05 |
| - | - | - | - |  | - | rs1531212 | 13951830 | upstream | 0.000559151 |
| - | - | - | - |  | - | rs895819 | 13947292 | upstream | 0.002824047 |
| LOC284454 | 0.034375023 | 19 | 13945329 | 1773 | unknown | rs35421192 | 13950221 | upstream | 2.31781E-06 |
| - | - | - | - |  | - | rs34597097 | 13950294 | upstream | 3.33579E-06 |
| - | - | - | - |  | - | 19:13950253 | 13950253 | upstream | 3.33579E-06 |
| - | - | - | - |  | - | rs7248169 | 13949751 | upstream | 3.38868E-06 |
| - | - | - | - |  | - | rs62122064 | 13950206 | upstream | 1.20859E-05 |
| - | - | - | - |  | - | rs1531212 | 13951830 | upstream | 0.000559151 |
| - | - | - | - |  | - | rs895819 | 13947292 | upstream | 0.002824047 |
| MIR23A | 0.034375023 | 19 | 13947400 | 72 | non-coding RNA | rs35421192 | 13950221 | upstream | 2.31781E-06 |
| - | - | - | - |  | - | rs34597097 | 13950294 | upstream | 3.33579E-06 |
| - | - | - | - |  | - | 19:13950253 | 13950253 | upstream | 3.33579E-06 |
| - | - | - | - |  | - | rs7248169 | 13949751 | upstream | 3.38868E-06 |
| - | - | - | - |  | - | rs62122064 | 13950206 | upstream | 1.20859E-05 |
| - | - | - | - |  | - | rs1531212 | 13951830 | upstream | 0.000559151 |
| - | - | - | - |  | - | rs895819 | 13947292 | upstream | 0.002824047 |
| UQCRC2 | 0.034375023 | 16 | 21964608 | 31000 | protein-coding gene | rs2945463 | 21960916 | upstream | 1.28578E-06 |
| - | - | - | - |  | - | rs2965807 | 21963203 | upstream | 1.28578E-06 |
| - | - | - | - |  | - | rs2945462 | 21959639 | upstream | 1.28578E-06 |
| - | - | - | - |  | - | rs9934359 | 21999124 | intronic | 1.52868E-06 |
| - | - | - | - |  | - | rs2967155 | 21984477 | intronic | 2.18966E-06 |
| - | - | - | - |  | - | rs2945464 | 21975415 | intronic | 2.24157E-06 |
| - | - | - | - |  | - | rs2967178 | 21970546 | intronic | 2.24157E-06 |
| - | - | - | - |  | - | 16:21997551 | 21997551 | intronic | 0.000175713 |
| - | - | - | - |  | - | rs8049791 | 21967307 | intronic | 0.00180098 |
| - | - | - | - |  | - | rs7200959 | 21981113 | intronic | 0.00180098 |
| - | - | - | - |  | - | rs4392055 | 21997834 | intronic | 0.00180098 |
| - | - | - | - |  | - | rs56036711 | 21990760 | intronic | 0.00180098 |
| - | - | - | - |  | - | rs6497563 | 21966869 | intronic | 0.00180098 |
| - | - | - | - |  | - | rs7196121 | 21966574 | intronic | 0.00180098 |
| - | - | - | - |  | - | rs77032576 | 21991449 | intronic | 0.00180098 |
